# Development of a CRISPR/SHERLOCK-Based Method for Rapid and Sensitive Detection of Selected Pospiviroids

**DOI:** 10.3390/v16071079

**Published:** 2024-07-04

**Authors:** Ying Zhai, Prabu Gnanasekaran, Hanu R. Pappu

**Affiliations:** 1 Department of Plant Pathology, Washington State University, Pullman, WA 99164, USA; 2 San Joaquin Valley Agricultural Sciences Center, USDA-ARS, Parlier, CA 93648, USA

**Keywords:** pospiviroid, CRISPR, Cas9, diagnosis, on-site detection, real-time RT-PCR

## Abstract

Pospiviroids infect a wide range of plant species, and many pospiviroids can be transmitted to potato and tomato. Pospiviroids continue to be a major production constraint as well as of quarantine concern for the movement of germplasm, and are regulated in several countries/regions. The USDA APHIS issued a federal order requiring all imported tomato and pepper seeds be certified free of six pospiviroids of quarantine significance. The six pospiviroids of quarantine interest include CLVd, PCFVd, PSTVd, TASVd, TCDVd, TPMVd. Currently, those six viroids are detected by real-time RT-PCR. CRISPR/Cas-based genome editing has been increasingly used for virus detection in the past five years. We used a rapid Cas13-based Specific High-sensitivity Enzymatic Reporter unLOCKing (SHERLOCK) platform for pospiviroid detection, determined the limits of detection and specificity of CRISPR-Cas13a assays. This platform combines recombinase polymerase amplification (RPA) with CRISPR and CRISPR-associated (CRISPR-Cas) RNA-guided endoribonuclease that is rapid and does not require expensive equipment, and can be adapted for on-site detection.

## 1. Introduction

Viroids are single-stranded, circular RNAs molecules with relatively small genomes of about 239–401 nt that infect only plants. Unlike viruses, viroids do not code for any protein. So far, 33 different viroid species have been characterized and grouped into two families, *Avsunviroidae* and *Pospiviroidae* [1]. The family *Pospiviroidae* was named from its type species, potato spindle tuber viroid (PSTVd). The *Pospiviroidae* contains five genera, *Pospiviroid*, *Hostuviroid, Apscaviroid*, *Cocadviroid and Coleviroid* [2]. Viroids in the family *Pospiviroidae* replicate in the nucleus using host DNA-dependent RNA polymerase II and their multimers are cleaved by host enzymes; In contrast, *Avsunviroidae* viroids replication takes place in chloroplasts. The nuclear-encoded chloroplast RNA polymerase transcribes the circular (+) RNA to a linear (−) strand concatemer, and the multimers are processed by ribozyme-mediated self-cleavage [3,4,5,6].

There are nine viroid species in the genus *Pospiviroid* [7,8], including PSTVd (type species), chrysanthemum stunt viroid, citrus exocortis viroid, columnea latent viroid (CLVd), iresine viroid 1, pepper chat fruit viroid (PCFVd), tomato apical stunt viroid (TASVd), tomato chlorotic dwarf viroid (TCDVd), tomato planta machoviroid (TPMVd), and portulaca latent viroid. These species are demarcated based on their total viroid genome sequence identities (less than 90%) and distinctive biological properties, particularly host range and symptoms [2,8]. Although PSTVd and TCDVd share more than 90% identity of genome sequences, their differences in host range and symptoms justify them as distinct species [9,10].

Pospiviroids can infect a wide range of plant species. All pospiviroids except iresine viroid 1 and the related portulaca latent viroid can be transmitted to potato and tomato, with similar symptoms observed under controlled conditions [11,12]. In addition to mechanical transmission [13], several pospiviroids were reported to be transmitted via pollen and seeds [14].

Pospiviroids continue to be a production constraint as well as a quarantine reason for new germplasm arriving in several countries and regions including the United States. The USDA APHIS issued a federal order requiring all imported tomato and pepper seeds be certified free of six pospiviroids of quarantine significance, or produced in countries where these pospiviroids are not known to occur. The six pospiviroids of quarantine interest include CLVd, PCFVd, PSTVd, TASVd, TCDVd, TPMVd.

Currently, viroids are detected by real-time RT-PCR. CRISPR/Cas-based genome editing has been increasingly used for virus detection in the past five years [15,16,17,18,19,20,21,22,23,24]. Different from Cas9, both Cas12 and Cas 13 proteins possess collateral or trans-cleavage activity. Cas12 is an endonuclease guided by crRNA to find the target DNA sequence, and then cuts both strands and the collateral DNA. Cas13 is an RNA-guided RNase which cleaves single-stranded and collateral RNAs. In both Cas12-based DNA endonuclease-targeted CRISPR trans reporter (DETECTR) [25] and Cas13-based Specific High-sensitivity Enzymatic Reporter unLOCKing (SHERLOCK) [26] systems, fluorescence signals can be generated via separation of the quencher from the reporter DNA/RNA by target recognition. In this work, we used a rapid SHERLOCK platform for pospiviroid detection. This platform combines recombinase polymerase amplification (RPA) with CRISPR and CRISPR-associated (CRISPR-Cas) RNA-guided endoribonuclease. It is highly specific in differentiating closely related pospiviroid pathogens, does not require expensive equipment, and can be adapted for on-site detection. To our knowledge, this is the first report of using SHERLOCK to detect pospiviroids of quarantine interest. 

## 2. Materials and Methods

### 2.1. Synthesis of RNA Transcripts

The complete genomes of CLVd (NC_003538.1), PCFVd (NC_011590.1), PSTVd (NC_002030.1), TASVd (MG132058.1), TCDVd (NC_000885.1) and TPMVd (NC_001558.1) were synthesized de novo and cloned into pET-3a vectors by GenScript (Piscataway, NJ, USA). The *in vitro* RNA transcription was carried out using a HiScribe™ T7 High Yield RNA Synthesis Kit (New England Biolabs, Ipswich, MA, USA) following the manufacturer’s protocol. Two pairs of PCR primers (pET3a-For-1: 5′-GCGTCCGGCGTAGAGGATCG-3′ & pET3a-Rev-1: 5′-GATCATGGCGACCACACCCGTC-3′; pET3a-For-2: 5′-ATCGGTGATGTCGGCGATATAG-3′ & pET3a-Rev-2: 5′-TACTTGGAGCCACTATCGACTAC-3′) were used to amplify the amplicons containing T7 RNA Polymerase promoter in the sense orientation. PCR products were purified with Monarch PCR & DNA Cleanup Kit (NEB, Ipswich, MA, USA) according to the protocol and used as templates for RNA synthesis. The reaction was set up with 1 μg purified PCR products, 2 μL ATP (100 mM), 2 μL GTP (100 mM), 2 μL UTP (100 mM), 2 μL CTP (100 mM), 2 μL 10× reaction buffer, and 2 μL T7 RNA Polymerase mix. The total volume was brought to 20 μL with nuclease-free water. After mixing the contents, the mixture was incubated at 37 °C for 2 h. 

### 2.2. Plant RNA Extraction

Total RNA was extracted from healthy and viroid-infected host plant seed using the RNeasy Plant Kit (Qiagen GmbH, Hilden, Germany). Briefly, about 100 mg of tissues per sample was grounded into fine powder with mortar and pestle in liquid nitrogen. Buffer RLT (450 µL) and 4.5 µL β-mercaptoethanol were added to the tissue powder. The lysate was transferred to a QIAshredder spin column and centrifuged for 2 min at 13,000 rpm. The flow-through supernatant was then transferred to a new microcentrifuge tube, and mixed with half volume of ethanol (96–100%). An aliquot (650 μL) of mixture was added to an RNeasy Mini spin column and centrifuged for 15 s at ≥8000× *g* (≥10,000 rpm). Buffer RW1 of 700 µL was added to the RNeasy spin column and centrifuged for 15 s at ≥8000× *g*. RNeasy spin column was washed with 500 µL of Buffer RPE twice. RNase-free water of 30–50 µL was added to the spin column and centrifuged to elute RNA. Healthy potato and pepper leaves were used as the negative control for PSTVd, and PCFVd detection, respectively. Healthy tomato leaves were used as the negative control for CLVd, TASVd, TCDVd, and TPMVd detection.

### 2.3. Design of RPA Primers and crRNA

Each viroid crRNA is composed of a direct repeat of LwaCas13a and a 28-nt spacer sequence specific to the pospiviroid. The 28-nt spacer sequence should target a specific amplicon. For example, the spacer sequence of PSTVd-crRNA1 should be reversely complementary to a 28-nt PSTVd internal sequence amplified by the RPA primer pair PSTVd-RPA-For-1 and PSTVd-RPA-Rev-3. Therefore, the PSTVd combination 1 includes PSTVd-RPA-For-1, PSTVd-RPA-Rev-3 and PSTVd crRNA-1. PSTVd combinations 1 (PSTVd-RPA-For-1 & PSTVd-RPA-Rev-3 + PSTVd crRNA-1) and 2 (PSTVd-RPA-For-2 & PSTVd-RPA-Rev-2 + PSTVd crRNA-2) were designed targeting regions 10–133 nt and 158–294 nt, respectively. PCFVd combinations 1 (PCFVd-RPA-For-1 & PCFVd-RPA-Rev-1 + PCFVd crRNA-1) and 2 (PCFVd-RPA-For-2 & PCFVd-RPA-Rev-2 + PCFVd crRNA-2) target regions 52–169 nt and 170–287 nt, respectively. CLVd combinations 1 (CLVd-RPA-For-3 & CLVd-RPA-Rev-3 + CLVd crRNA) and 2 (CLVd-RPA-For-3 & CLVd-RPA-Rev-4 + CLVd crRNA) share the same crRNA but have different RPA primer pairs. TASVd combinations 1 (TASVd-RPA-For-2 & TASVd-RPA-Rev-2 + TASVd crRNA) and 2 (TASVd-RPA-For-2 & TASVd-RPA-Rev-5 + TASVd crRNA) also share the same crRNA but have different RPA primer pairs. Both TCDVd (TCDVd-RPA-For-1 & TCDVd-RPA-Rev-1 + TCDVd crRNA) and TPMVd (TPMVd-RPA-For-1 & TPMVd-RPA-Rev-1 + TPMVd crRNA) only have one combination each (Table 1). Two forward and four reverse primers for PSTVd, three forward and four reverse primers for PCFVd, three forward and five reverse primers for CLVd, three forward and six reverse primers for TASVd, two forward and two reverse primers for TCDVd, and two forward and two reverse primers for TPMVd were designed. Notably, not all RPA primers worked well for this project. Each combination was used for both monoplex (RPA primers and crRNA were used to test their designated target pospiviroid) and specificity (RPA primers and crRNA were used to test all six pospiviroids) reactions.

### 2.4. Recombinase Polymerase Amplification (RPA)

RPA primers were designed based on genome sequences of the six pospiviroids, following the guidelines of 100–140 nt amplicon sizes, 54–67 °C primer melting temperatures, 30–35 nt primer lengths, and a T7 RNA polymerase promoter sequence (5′-GAAATTAATACGACTCACTATAGGG-3′, 25 nt) appended to the 5′ end of one primer to allow T7 transcription. RPA reactions were carried out using Twist Amp^TM^ Basic Kit (TwistDx, Maidenhead, UK) following the manufacturer’s protocol. The RPA reaction was set up as follows: 5.9 μL resuspended RPA solution, 0.5 μL RPA forward primer (10 μM), 0.5 μL RPA reverse primer (10 μM), 0.2 μL ProtoScript RT (100,000 U/mL), 1.4 μL Nuclease-free water, 1 μL RNA sample, and 0.5 μL MgOAc (280 mM). RPA reactions were performed at 39 °C for 20 min.

### 2.5. Nucleic Acid Detection Using Cas13a

The crRNAs were designed complementary to the genome sequence of pospiviroids. For the detection using LwaCas13a, the complete crRNA contains a direct repeat of LwaCas13a (5′-GAUUUAGACUACCCCAAAAACGAAGGGGACUAAAAC-3′, DR) and a 28-nt spacer sequence specific to each pospiviroid. Each detection assay was performed with 1 μL purified LwaCas13a protein (63.3 μg/mL), 0.2 μL HEPES (pH6.8, 1M), 1.8 μL MgCl_2_ (50 mM), 0.4 μL rNTP (25 mM each), 0.063 μL T7 RNA Polymerase (20 U/μL), 0.5 μL crRNA (10 ng/μL), 0.5μL Poly U Reporter (10 μM), 1 μL RPA product, and 4.04 μL nuclease-free water. Reactions were set up at 37 °C for 30 min. Results were visually documented first using handheld fluorometers in the dark, and then quantified with a BioTek Synergy Neo2 Hybrid Multimode Reader. The 10 μL products were mixed with 190 μL double-distilled water, and then aliquoted to two wells of 96-well black microtiter plate (Greiner Bio-One, Monroe, NC, USA). 

### 2.6. Comparative Sensitivity Analysis between Real-Time RT-PCR and CRISPR-Cas13 Based-Detection

Serial dilutions of synthetic pospiviroids RNA transcripts were used to compare the sensitivity of CRISPR-Cas13 and real-time RT-PCR [27]-based detection. RNA concentrations and purity were measured with a NanoDrop One C Spectrophotometer (Thermo Fisher, Waltham, MA, USA). cDNA synthesis was performed using Bio-Rad iScript Reverse Transcription Supermix (Bio-Rad, Hercules, CA, USA). Bio-Rad SYBR Green Supermix, CFX96 Real-Time System, and the CFX Manager software were used for the quantification of pospiviroids gene expression levels. 

## 3. Results 

### 3.1. Detection of Pospiviroids with CRISPR-Cas13a

**Figure 1:**
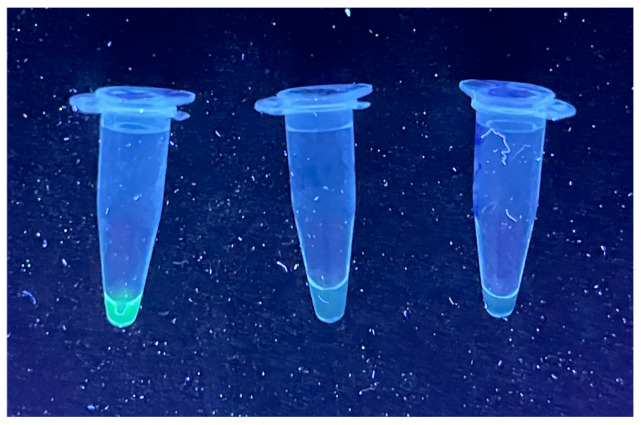
Visualization of fluorescence resulting from potato spindle tuber viroid (PSTVd) detection. From left to right, PSTVd RNA, water control, and healthy host RNA samples.

Although 37 °C was reported as the optimal reaction temperature, RPA reactions were performed at 39 °C for 20 min. as this temperature yielded the strongest amplification. The RPA amplicons were cloned into pGEM-T Easy vector and was confirmed by sequencing. The RPA products were used as the template for the Cas13a-based detection step. Green fluorescence was observed from PSTVd RNA transcript under UV light using PSTVd combination 1. There were no fluorescence signals with water and healthy-potato RNA controls (Figure 1). Similar results were obtained from the remaining five pospiviroids RNA transcripts using their specific combinations. The fluorescence density was also measured by fluorescence plate reader for quantification purposes. Fluorescence value was 5000–7000 with about 10ng viroid RNA transcript, while 400–600 was with healthy host RNA (negative control). Using this platform, we also tested two negative and three PSTVd-positive plant RNA samples previously confirmed of their infection status by real-time RT-PCR, and got consistent results for all the samples.

### 3.2. The Limits of Detection (LOD) of Monoplex and Specificity CRISPR-Cas13a Assays

**Figure 2:**
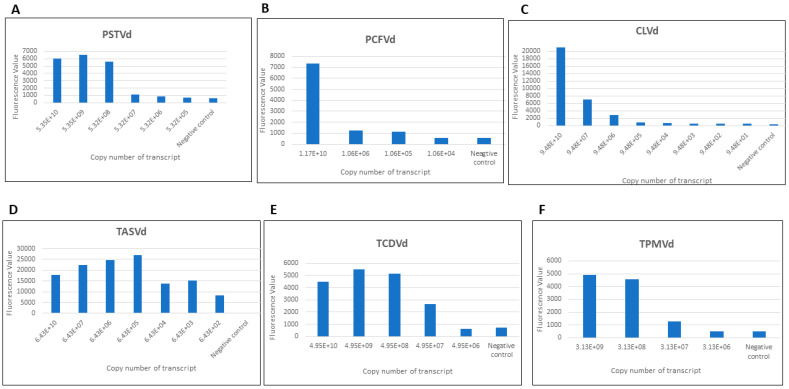
Sensitivity analysis of monoplex CRISPR-Cas13a based detection of six pospiviroids (**A**–**F**). All experiments were repeated three times with similar results obtained. X-axis represents the copy number of serially diluted RNA transcript. Y-axis represents the fluorescence value.

Pospiviroid RNA transcripts were diluted to determine LOD. The monoplex sensitivity assay showed that the CRISPR-Cas13a based detection had variable LOD with the six pospiviroids, with copy numbers of 5.32E+7 for PSTVd (via PSTVd combination 1), 1.06E+5 for PCFVd (via PCFVd combination 1), 9.48E+6 for CLVd (via CLVd combination 1), 6.43E+2 for TASVd (via TASVd combination 1), 4.62E+7 for TCDVd (via TCDVd combination), and 3.13E+7 for TPMVd (via TPMVd combination) (Figure 2).

**Figure 3:**
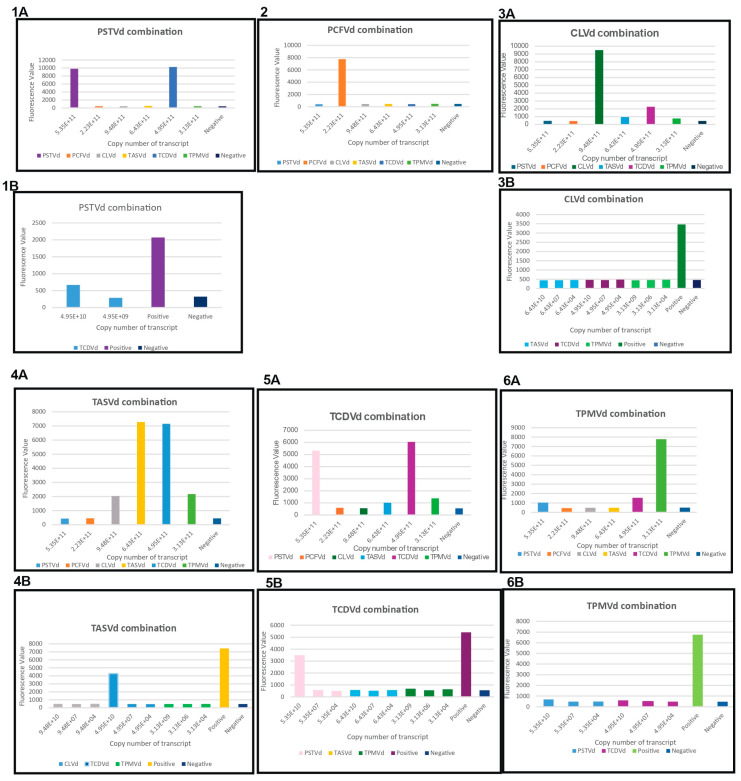
Specificity assay of six pospiviroids and their LOD. (**1A**) specificity test on all six pospiviroid RNA transcripts with high copy number using PSTVd combination 1; (**1B**) specificity test on serially diluted TCDVd transcripts using PSTVd combination 1. PSTVd RNA in 1A was used as a positive control in 1B. Healthy plant RNA was used as a negative control; (**2**) specificity test on all six pospiviroid RNA transcripts with high copy number using PCFVd combination 1; No dilution of other pospiviroids was performed for PCFVd combination 1 since it can specifically detect PCFVd. (**3A**) specificity test on all six pospiviroid RNA transcripts with high copy number using CLVd combination 1; (**3B**) specificity test on serially diluted TASVd, TCDVd and TPMVd transcripts using CLVd combination 1. CLVd RNA in 3A was used as a positive control in 3B. Healthy plant RNA was used as a negative control; (**4A**) specificity test on all six pospiviroid RNA transcripts with high copy number using TASVd combination 1; (**4B**) specificity test on serially diluted CLVd, TASVd, TCDVd and TPMVd transcripts using TASVd combination 1. TASVd RNA in 4A was used as a positive control in 4B. Healthy plant RNA was used as a negative control; (**5A**) specificity test on all six pospiviroid RNA transcripts with high copy number using TCDVd combination; (**5B**) specificity test on serially diluted PSTVd, TASVd, and TPMVd transcripts using TCDVd combination. TCDVd RNA in 5A was used as a positive control in 5B. Healthy plant RNA was used as a negative control; (**6A**) specificity test on all six pospiviroid RNA transcripts with high copy number using TPMVd combination; (**6B**) specificity test on serially diluted PSTVd, TCDVd transcripts using TPMVd combination. TPMVd RNA in 6A was used as a positive control in 6B. Healthy plant RNA was used as a negative control. All experiments were repeated three times with similar results obtained. X-axis represents the copy number of RNA transcript. Y-axis represents the fluorescence value.

For specificity assay, each combination of RPA primers and its crRNA targeting PSTVd, PCFVd, CLVd, TASVd, TCDVd or TPMVd were used to test all six pospiviroid RNA transcripts with sufficiently high copy numbers (the first column of each panel in Figure 2A–F). Their LODs were then determined using serially diluted RNA transcripts (Figure 3). For example, fluorescence signals were quantified for reactions using PSTVd combination 1 and each of the six viroids’ transcripts as templates. Transcripts with high copy numbers were used to identify potential cross reaction targets, and then serial dilutions were performed to determine the LODs. Based on our specificity reaction results (Table 2 and Figure 3), PSTVd combination 1 detected both PSTVd and TCDVd (LOD 4.95E+10). PCFVd combination 1 specifically detected PCFVd. CLVd combination 1 detected CLVd, TASVd (LOD 6.43E+11), TCDVd (LOD 4.95E+11), and TPMVd (LOD 3.13E+11). TASVd combination 1 detected TASVd, CLVd (LOD 9.48E+11), TCDVd (LOD 4.95E+10), and TPMVd (LOD 3.13E+11). The TCDVd combination detected TCDVd, PSTVd (LOD 5.35E+10), TASVd (LOD 6.43E+11), and TPMVd (LOD 3.13E+11). The TPMVd combination detected TPMVd, PSTVd (LOD 5.35E+11), and TCDVd (LOD 4.95E+11). In addition to its specific target, except PCFVd combination, the other five combinations also detected at least one other viroids at much higher LODs, which might be due to the extremely high homology shared by the six viroids.

### 3.3. Comparative Sensitivity Analysis between Real-Time RT-PCR and CRISPR-Cas13a Based-Detection

**Figure 4:**
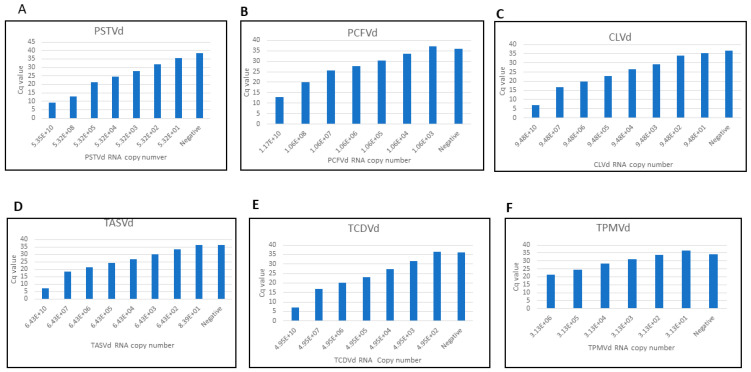
Sensitivity analysis of real-time RT-PCR detection of six pospiviroids (**A**–**F**). All experiments were repeated three times with similar results obtained. X-axis represents the copy number of serially diluted RNA transcript. Y-axis represents the Cq value.

**Figure 5:**
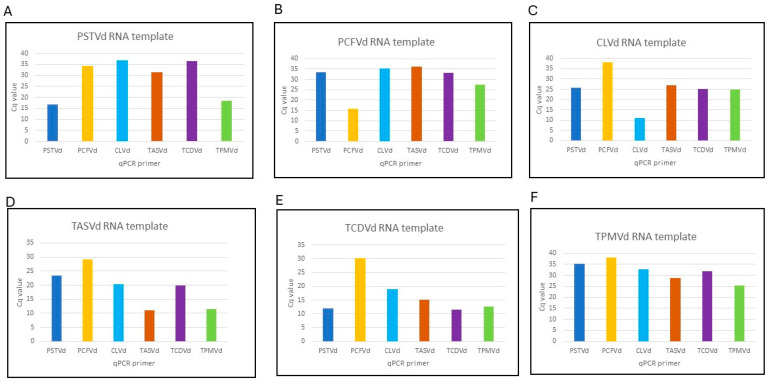
Specificity analysis of real-time RT-PCR primers (**A**–**F**). The Cq value of all real-time RT-PCR primers were more than 39 when water was used as the control. All experiments were repeated three times with similar results obtained. X-axis represents the RNA transcript. Y-axis represents the Cq value.

Serial dilutions of synthetic RNA transcripts of pospiviroids were made to compare the sensitivity of CRISPR-Cas13 and real-time RT-PCR-based detection. Real-time RT-PCR primers for pospiviroids genes are listed in Table 3. Pospiviroid genomes are closely related, therefore, it is difficult to design real-time RT-PCR primers that are specific to individual pospivirod. The real-time RT-PCR sensitivity assay showed lower LOD than CRISPR-Cas13a for most pospiviroids, with copy number 5.32E+03 for PSTVd, 1.06E+06 for PCFVd, 9.48E+04 for CLVd, 6.43E+04 for TASVd, 4.95E+04 for TCDVd and 3.13E+04 for TPMVd (Figure 4). The specificity of real-time RT-PCR primers used was also tested (Table 4, Figure 5). PSTVd real-time RT-PCR primers detected not only PSTVd RNA, but also CLVd, TASVd, and TCDVd RNA templates. PCFVd real-time RT-PCR primers specifically detected only PCFVd RNA template. CLVd real-time RT-PCR primers, besides CLVd RNA also detected TASVd and TCDVd RNA templates. TASVd-specific real-time RT-PCR primers, besides TASVd RNA, also detected CLVd, TCDVd and TPMVd RNA templates. TCDVd-specific real-time RT-PCR primers, besides TCDVd RNA, also detected CLVd and TASVd RNA. TPMVd real-time RT-PCR primers, besides TPMVd, detected all other five pospiviroids RNA templates. The Cq value of all real-time RT-PCR primers was more than 39 when using healthy host RNA as the control. Among all real-time RT-PCR primers, those targeting PCFVd had the highest specificity and amplified only PCFVd, whereas TPMVd real-time RT-PCR primers can be used as a universal primer set to detect all the six viroids. Overall, neither real-time RT-PCR- nor CRISPR-Cas13a-based detection has high LODs or viroid specificity.

## 4. Discussion

Viroids are the smallest plant pathogens and consist of naked, circular single-stranded RNA and do not code for any proteins [28]. Consequently, serodiagnostic techniques for viroid detection are not applicable except for recently reported polyclonal antibodies against PSTVd RNA [29]. Traditional viroid detection methods such as biological indexing are used for the identification of causal agents [30]. Nucleic acid-based techniques including RT-PCR, real-time RT-PCR, RT loop-mediated isothermal amplification (RT-LAMP), isothermal and chimeric primer-initiated amplification of nucleic acids (ICAN), micro- and macro arrays, next-generation sequencing (NGS), and CRISPR-Cas12/13 are being used for detection. These methods offer faster, more sensitive, more reliable, and on-site testing options for detecting viroid infections [31].

There have been several reports of detection of plant virus/viroid using the CRISPR-Cas12/13 platform. Li et al. [32] developed a plasmonic CRISPR Cas12a assay to visually detect the colorimetric signal emitted by grapevine red-blotch viral infection in the vineyard. Jiao et al. [19] used a similar CRISPR/Cas12a-RT-RPA visual platform for detection of multiple apple-infecting viruses and the apple scar skin viroid (ASSVd). Coupled with either RT-RPA or LAMP, the CRISPR–Cas12a module can be used to detect plant RNA [33] and DNA [34] viruses, respectively. Furthermore, the CRISPR–Cas12a platform is sensitive enough for species-specific detection of similar viruses, such as differential diagnosis of tobamoviruses tomato mosaic virus and tomato brown rugose fruit virus [35]. Marqués et al. [18] applied the CRISPR-Cas12a and CRISPR-Cas13a/d systems for indirect detection of viral DNA amplicons and direct diagnosis of viral RNAs, respectively. Among these reports, the CRISPR-Cas12a is much more popular with only one [18] involving CRISPR-Cas13a, which is the basis of SHERLOCK. Moreover, CRISPR/Cas-based plant viroid detection is reported only for ASSVd [19] with the sensitivity of ASSVd assay being significantly lower than those for other plant viruses when using the same CRISPR/Cas12a-based platform.

**Figure 6:**
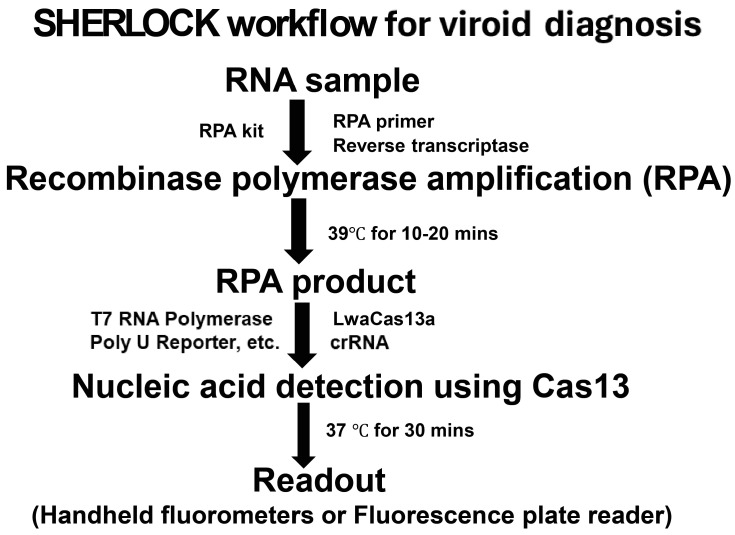
SHERLOCK workflow for viroid diagnosis.

Here, we used the SHERLOCK platform for pospiviroid detection (Figure 6), although with a lower detection sensitivity, it has the advantage of on-site testing compatibility. The short sequence length and high homology made it difficult to establish an ideal detection method, except for the high-cost and time-consuming direct sequencing approach.

Among all combinations tested, PCFVd combination 1 was the only one that was specific in all the methods used. Although most of the RPA primer and crRNA combinations did not show high specificity when the viroid concentrations were high, their specificities increased with the reduction of non-target viroid concentrations. For example, PSTVd combination 1 was PSTVd-specific as long as TCDVd copy number did not reach at least 4.95E+10. CLVd combination 1 detected viroids other than CLVd only when the copy numbers of TASVd, TCDVd, or TPMVd reached E+11 level. Similarly, TASVd combination 1 was specific to TASVd detection with relatively low copy numbers of CLVd, TCDVd, and TPMVd. Insufficient (copy number thresholds vary from E+10 to E+11) PSTVd, TASVd, or TPMVd did not interfere with the specific targeting of TCDVd combination to TCDVd either. In addition to TPMVd, the TPMVd combination was able to detect E+11 level PSTVd and TCDVd. In contrast, the copy numbers required for anticipated viroid detection using these combinations ranged from E+2 to E+7. Considering that viroid copy numbers in plant hosts can rarely reach E+10 to E+11 levels, our SHERLOCK platform may have adequate specificity in real-world viroid detection.

Due to the different nature of real-time RT-PCR and CRISPR-Cas13a reactions, the SHERLOCK platform had lower sensitivity as compared to the more traditional real-time RT-PCR approach. Real-time RT-PCR assays need to be performed in the lab with real-time PCR machine, and the data (Cq value) can only be obtained from the equipment. In contrast, the CRISPR-Cas13a-based viroid detection system can be adapted to on-site application with no need of costly instruments, and the output is visible under portable UV light in the field, which are critical advantages over real-time RT-PCR. In the future, SHERLOCK viroid diagnosis platform needs further refinement to improve its sensitivity to a level comparable to that of real-time RT-PCR. In addition, expanding the LOD gaps between targeted and non-specific viroids would further increase the specificity of the platform. On the other hand, it’s difficult to apply the current multiple viroid detection system (results from specificity assay) for diagnosis purpose due to the high LOD values required, whereas, narrowing viroid LOD gaps and reducing their absolute LOD values would facilitate detection of up to all six regulated viroids. Owing to their relatively high sensitivity and fewer steps, PCR-based viroid detection protocols [36,37,38,39] may not be readily replaced by the SHERLOCK viroid detection platform. 

## Figures and Tables

**Table 1:** RPA primers and crRNAs used in this study.

Combination	RPA Primer	Sequence (5′-3′)	crRNA Using LwaCas13a(5′-3′)	Target
PSTVd combination 1	PSTVd-RPA-For-1	**GAAATTAATACGACTCACTATAGGG**ACTAAACTCGTGGTTCCTGTGGTTCACAC	GAUUUAGACUACCCCAAAAACGAAGGGGACUAAAA*CCCCUGAAGCGCUCCUCCGAGCCGCCUUC*	PSTVd
	PSTVd-RPA-Rev-3	CTCCCCACCGTCCTTATTGCCAGTTCGCT		
PSTVd combination 2	PSTVd-RPA-For-2	**GAAATTAATACGACTCACTATAGGG**AGTAATTCCCGCCGAAACAGGGTTTTCAC	GAUUUAGACUACCCCAAAAACGAAGGGGACUAAAA*CAGGGGGCGAGGGGUGGUCCUGCGGGCGC*	
	PSTVd-RPA-Rev-2	TTCTCGGGAGCTTCAGTTGTTTCCACCGGGTA		
PCFVd combination 1	PCFVd-RPA-For-1	**GAAATTAATACGACTCACTATAGGG**TAGGGAAAAGAAAGGGGAAGCAAGCATCTC	GAUUUAGACUACCCCAAAAACGAAGGGGACUAAAA*CCUUCUCCGCCCGGUCUGUCCAGGUUUCC*	PCFVd
	PCFVd-RPA-Rev-1	CTGCTGGGATTACTCCTGTCAGAAGACGGT		
PCFVd combination 2	PCFVd-RPA-For-2	**GAAATTAATACGACTCACTATAGGG**AAACAGGGTTTTCACCCTTCCTTTCTTCG	GAUUUAGACUACCCCAAAAACGAAGGGGACUAAAA*CGUGCGCGAGAAGGCCGACGCGGACCGGU*	
	PCFVd-RPA-Rev-2	GCACCTCTGTCAGTTGTATCCACCGGGTAG		
CLVd combination 1	CLVd-RPA-For-3	**GAAATTAATACGACTCACTATAGGG**CCATGCAAAAGAAAAAAGAACGGGAGGG	GAUUUAGACUACCCCAAAAACGAAGGGGACUAAAA*CGCUCGGUCUGAGUUGCCCCGGGGCUCCU*	CLVd
	CLVd-RPA-Rev-3	CTCCTGTCTGAACAGGGCAACGCCCTCGAC		
CLVd combination 2	CLVd-RPA-For-3	**GAAATTAATACGACTCACTATAGGG**CCATGCAAAAGAAAAAAGAACGGGAGGG		
	CLVd-RPA-Rev-4	AGGAAGGGTGAAAACCCTGTTTCAGCTGGG		
TASVd combination 1	TASVd-RPA-For-2	**GAAATTAATACGACTCACTATAGGG**CAGCTGAAACAGGGTTTTCACCCTTCC	GAUUUAGACUACCCCAAAAACGAAGGGGACUAAAA*CGGCGAGCGCCGAAGACCUUCCGGCGAGA*	TASVd
	TASVd-RPA-Rev-2	CCGTGGAGTCGAAGCTTCAGTTGTTTCC		
TASVd combination 2	TASVd-RPA-For-2	**GAAATTAATACGACTCACTATAGGG**CAGCTGAAACAGGGTTTTCACCCTTCC		
	TASVd-RPA-Rev-5	AGATAGAGAAAAAGAGCCGTGGAGTCGAAGC		
TCDVd combination	TCDVd-RPA-For-1	**GAAATTAATACGACTCACTATAGGGG**TGGTTCCTGTGGTTCACACCTGACCTCC	GAUUUAGACUACCCCAAAAACGAAGGGGACUAAAA*CGUUUCCCCGGGGAUCCCUGAAGCGCUCC*	TCDVd
	TCDVd-RPA-Rev-1	CCTGTTTCGCCTTCCACAAGCTCCCTGC		
TPMVd combination	TPMVd-RPA-For-1	**GAAATTAATACGACTCACTATAGGGG**TGGTTCCTGTGGTTCACACCTGACCTCC	GAUUUAGACUACCCCAAAAACGAAGGGGACUAAAA*CGGGAUCCCUGAAGCGCUCCUUUGGCCGC*	TPMVd
	TPMVd-RPA-Rev-1	CAGCGGGGATTACTCCTGTCTGGGAGAC		

Note: The T7 RNA polymerase promoter sequence (5′-**GAAATTAATACGACTCACTATAGGG**-3′) was indicated in bold. The spacer sequence targeting each viroid was italicized.

**Table 2:** Specificity CRISPR-Cas13a assay of pospiviroids and healthy host samples. The copy numbers of PSTVd, PCFVd, CLVd, TASVd, TCDVd and TPMVd transcripts are 5.35E+11, 2.34E+11, 9.48E+11, 6.43E+11, 4.95E+11 and 3.13E+11, respectively. Healthy host RNAs were isolated and used for negative control. For PSTVd, pepper was used as the host. For PSTVd, potato was used as the host. For CLVd, TASVd, TCDVd and TPMVd, tomato was used as the host. The RNA concentration from healthy hosts was 50 ng/μL. “+” indicates viroid transcript detection by certain combination. “-“ indicates no transcript detection by certain combination.

	PSTVd Transcript	PCFVd Transcript	CLVd Transcript	TASVd Transcript	TCDVd Transcript	TPMVd Transcript	Healthy Host
PSTVd combination 1	+	-	-	-	+	-	-
PCFVd combination 1	-	+	-	-	-	-	-
CLVd combination 1	-	-	+	+	+	+	-
TASVd combination 1	-	-	+	+	+	+	-
TCDVd combination	+	-	-	+	+	+	-
TPMVd combination	+	-	-	-	+	+	-

**Table 3:** List of real-time RT-PCR primers designed and used for the detection of viroids.

Real-Time RT-PCR Primer	Sequence (5′-3′)
PSTVd-For	CTGGAGCGAACTGGCAATAA
PSTVd-Rev	CCGAAGAAAGGAAGGGTGAAA
PCFVd-For	CTTTCTTCGGGTTTCCTTCCT
PCFVd-Rev	AAGCACCTCTGTCAGTTGTATC
CLVd-For	ACCCTTCCTTTCTTCTGGTTTC
CLVd-Rev	CCGGAGACCAAGCTAAGATAGA
TASVd-For	ACCCTTCCTTTCTTCTGGTTTC
TASVd-Rev	GGAGTCGAAGCTTCAGTTGTT
TCDVd-For	TCCTTTCTTCTGCGGTTTCC
TCDVd-Rev	TCGGGAGCTTCAGTTGTTTC
TPMVd-For	CTTCCTTTCTTCGGGTTTCCT
TPMVd-Rev	TGGGAGCTTCAGTTGTTTCC

**Table 4:** Sensitivity real-time RT-PCR detection of six pospiviroids. The copy numbers of PSTVd, PCFVd, CLVd, TASVd, TCDVd and TPMVd transcripts were 5.35E+11, 2.34E+11, 9.48E+11, 6.43E+11, 4.95E+11 and 3.13E+11, respectively. Healthy host RNAs were isolated and used for negative control. For PSTVd test, pepper was used as the host. For PSTVd test, potato was used as the host. For CLVd, TASVd, TCDVd and TPMVd tests, tomato was used as the host. The RNA concentration from healthy hosts was 50 ng/μL. “+” indicates viroid transcript detection by certain real-time RT-PCR primers. “-“ indicates no transcript detection by certain real-time RT-PCR primers.

	PSTVd Transcript	PCFVd Transcript	CLVd Transcript	TASVd Transcript	TCDVd Transcript	TPMVd Transcript	Healthy Host
PSTVd real-time RT-PCR primers	+	-	+	+	+	-	-
PCFVd real-time RT-PCR primers	-	+	-	-	-	-	-
CLVd real-time RT-PCR primers	-	-	+	+	+	-	-
TASVd real-time RT-PCR primers	-	-	+	+	+	+	-
TCDVd real-time RT-PCR primers	-	-	+	+	+	-	-
TPMVd real-time RT-PCR primers	+	+	+	+	+	+	-

## Data Availability

The data presented in this study are available on request from the corresponding author.
